# An American Nurse's View

**Published:** 1919-01-04

**Authors:** 


*To the Editor of* The Hospital.

### An American Nurse's View.

*To the Editor of* The Hospital.

Sir,—*Re* "Ierne's" article, "Wake Up, College of Nursing!" may a "rank outsider" (an American nurse) say a word on the subject of the cry, "Have patience, and it will all come right"?

Let me remind the "patient ones" that "any virtue carried to excess becomes a vice," and it seems to me thirty-five years (I believe that is the length of time British nurses have "waited with patience" for registration) is long enough. The thing for them to do now is to "get up and hustle for it." Build from the bottom, set a standard (not too high) for registration, pass a Bill, and "build up" to lectures, examinations, scholarships, and all the rest.

What I would like to know is—Does the "British Nurse" want registration? If she does, the matter is in her own hands. Or "is she a slave to 'convention hugging her chains'"? Nurses never were agitators, etc., etc. Neither were the victorious armies of Britain and United States of America "soldiers" (by profession), but they fought when occasion arose. When the majority consider a thing "worth while" it is bound to go through. Do the majority of British nurses wish changes—better salaries, shorter hours; in fact, time to live as well as exist; or are they content to let "well enough" alone? I wonder!

A U.S.A. Registered Nurse.

